# P-627. Modeling the Clinical and Economic Impact of Increasing Varicella Vaccination Coverage in Panama

**DOI:** 10.1093/ofid/ofae631.825

**Published:** 2025-01-29

**Authors:** Colleen Burgess, Salome Samant, Luciana Hirata, Cintia I Parellada, Dora Estripeaut, Manjiri D Pawaskar, John C Lang

**Affiliations:** Merck & Co., Inc., Rahway, NJ, USA, Rahway, New Jersey; Merck & Co., Inc., Kenilworth, New Jersey; MSD Brazil, São Paulo, SP, Brazil, São Paulo, Sao Paulo, Brazil; MSD Brazil, São Paulo, Sao Paulo, Brazil; Hospital del Niño, Panama City, Panama, Panama, Panama, Panama; Merck & Co., Inc., Kenilworth, New Jersey; Merck Canada Inc., Kirkland, QC, Canada, Kirkland, Quebec, Canada

## Abstract

**Background:**

Panama implemented two-dose universal varicella vaccination (UVV) in 2018. First-dose vaccination coverage rates (VCRs) declined from 91% (2018) to 70% (2021) during the COVID-19 pandemic. We quantified the clinical and economic impact of increasing UVV VCRs over a 10-year time horizon.

**Figure:**
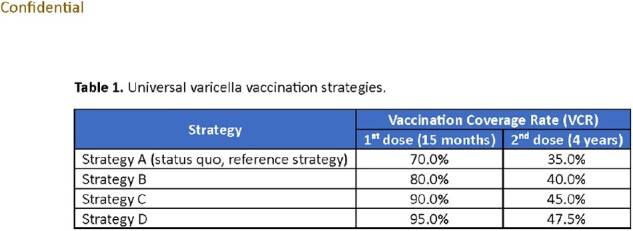

**Methods:**

A previously published age-structured dynamic transmission model was adapted to Panama using country-specific demographic, healthcare resource use, cost, and epidemiological data or comparable proxy. Four UVV strategies (1^st^ dose: 15 months; 2^nd^ dose: 4 years) were evaluated, with 1^st^ dose VCR assumed to be (A) 70% (status quo), (B) 80%, (C) 90%, and (D) 95% for the strategies A-D (Table 1). Second dose VCR was assumed to be 50% of first dose VCR. Outcomes were evaluated over a 10-year time horizon (2022-2031) and included cumulative varicella cases, outpatient cases, hospitalizations, deaths, payer (direct) costs, and societal (direct and indirect) costs. Costs were reported in 2023 USD with 3% annual discounting.

**Figure:**
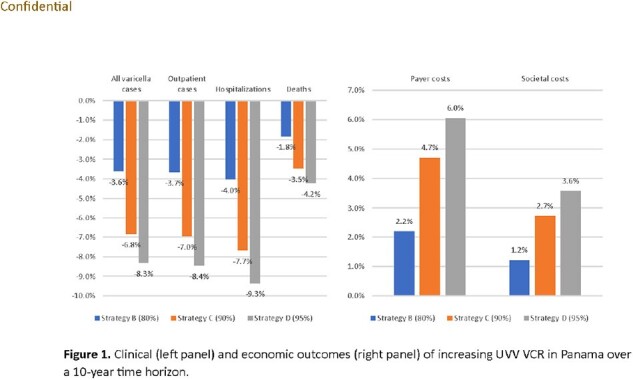

**Results:**

Under the status quo (strategy A), we estimated 232,381 cumulative varicella cases, 159,123 outpatient cases, and 7,320 hospitalizations over 10 years, resulting in total payer and societal costs of $29,335,329 and $36,893,235, respectively. Increasing 1^st^ dose/2^nd^ dose VCR from 70%/35% (Strategy A) to 80%/40% (Strategy B) will avert approximately 3.6% of varicella cases, 4% of hospitalizations, and 1.8% of deaths. Increasing VCR to 90%/45% (Strategy C) will avert 6.8% of all cases, 7.7% of hospitalizations, and 3.5% of deaths compared to reference Strategy A. The impact of increasing VCRs to 95%/47.5% (Strategy D) resulted in a further reduction in varicella cases by about 20% more than Strategy C. Strategies B-D increased payer costs by 2%-6% ($0.02-$0.04 per person per year [PPPY]) and societal costs by 1%-4% ($0.01-$0.03 PPPY) over the 10-year time horizon versus reference strategy A (Figure 1).

**Conclusion:**

Increasing UVV VCRs compared to the status quo will result in improved clinical outcomes with marginal increases in payer and societal costs.

**Disclosures:**

**Colleen Burgess, MS**, Merck & Co., Inc., Rahway, NJ, USA: Contractor **Salome Samant, MBBS, MPH**, Merck & Co., Inc., Rahway, NJ, USA: Employee- earned salary and own stock|Merck & Co., Inc., Rahway, NJ, USA: Stocks/Bonds (Public Company) **Luciana Hirata, PhD**, Merck & Co., Inc., Rahway, NJ, USA.: I am an employee of MSD subsidiary of Merck & Co., Inc., Rahway, NJ, USA. and may hold stock or stock options in Merck & Co., Inc. **Cintia I. Parellada, MD, PhD**, Merck & Co., Inc: Employee|Merck & Co., Inc: Stocks/Bonds (Private Company) **Manjiri D. Pawaskar, PhD**, merck: employee|merck: Stocks/Bonds (Public Company) **John C. Lang, PhD, MSc, MSc, BSc**, Merck & Co., Inc.: Stocks/Bonds (Private Company)|Merck Canada Inc.: Employee

